# Correlation between tissue Doppler-derived left ventricular systolic velocity (S’) and left ventricle ejection fraction in sepsis and septic shock: a retrospective cohort study

**DOI:** 10.1186/s40560-023-00678-z

**Published:** 2023-07-03

**Authors:** Sanchit Chawla, Ryota Sato, Abhijit Duggal, Mahmoud Alwakeel, Daisuke Hasegawa, Dina Alayan, Patrick Collier, Filippo Sanfilippo, Michael Lanspa, Siddharth Dugar

**Affiliations:** 1 Department of Critical Care Medicine, Respiratory Institute, Cleveland Clinic, Cleveland, OH USA; 2 Cleveland Clinic Lerner College of Medicine, Case Western University Reserve University, Cleveland, OH USA; 3 Department of Internal Medicine, Mount Sinai Beth Israel, New York, NY USA; 4 Department of Medicine, Cleveland Clinic, Cleveland, OH USA; 5 Department of Cardiovascular Medicine, Heart, Vascular, and Thoracic Institute, Cleveland Clinic, Cleveland, OH USA; 6 Anaesthesiology and Intensive Care, University of Catania, Catania, Italy; 7 Policlinico-San Marco University Hospital, Catania, Italy; 8 Critical Care Echocardiography Service, Intermountain Medical Center, Murray, UT USA; 9 Division of Pulmonary and Critical Care Medicine, University of Utah, Salt Lake City, UT USA

**Keywords:** Sepsis, Shock, Tissue Doppler Imaging, Left ventricle systolic dysfunction, Correlation, Mitral atrioventricular plane, ICU mortality

## Abstract

**Background:**

Tissue Doppler-derived left ventricular systolic velocity (mitral S’) has shown excellent correlation to left ventricular ejection fraction (LVEF) in non-critically patients. However, their correlation in septic patients remains poorly understood and its impact on mortality is undetermined. We investigated the relationship between mitral S’ and LVEF in a large cohort of critically-ill septic patients.

**Methods:**

We conducted a retrospective cohort study between 01/2011 and 12/2020. All adult patients (≥ 18 years) who were admitted to the medical intensive care unit (MICU) with sepsis and septic shock that underwent a transthoracic echocardiogram (TTE) within 72 h were included. Pearson correlation test was used to assess correlation between average mitral S’ and LVEF. Pearson correlation was used to assess correlation between average mitral S’ and LVEF. We also assessed the association between mitral S’, LVEF and 28-day mortality.

**Results:**

2519 patients met the inclusion criteria. The study population included 1216 (48.3%) males with a median age of 64 (IQR: 53–73), and a median APACHE III score of 85 (IQR: 67, 108). The median septal, lateral, and average mitral S’ were 8 cm/s (IQR): 6.0, 10.0], 9 cm/s (IQR: 6.0, 10.0), and 8.5 cm/s (IQR: 6.5, 10.5), respectively. Mitral S’ was noted to have moderate correlation with LVEF (*r* = 0.46). In multivariable logistic regression analysis, average mitral S’ was associated with an increase in both 28-day ICU and in-hospital mortality with odds ratio (OR) 1.04 (95% CI 1.01–1.08, *p* = 0.02) and OR 1.04 (95% CI 1.01–1.07, *p* = 0.02), respectively.

**Conclusions:**

Even though mitral S’ and LVEF may be related, they are not exchangeable and were only found to have moderate correlation in this study. LVEF is U-shaped, while mitral S’ has a linear relation with 28-day ICU mortality. An increase in average mitral S’ was associated with higher 28-day mortality.

**Graphical Abstract:**

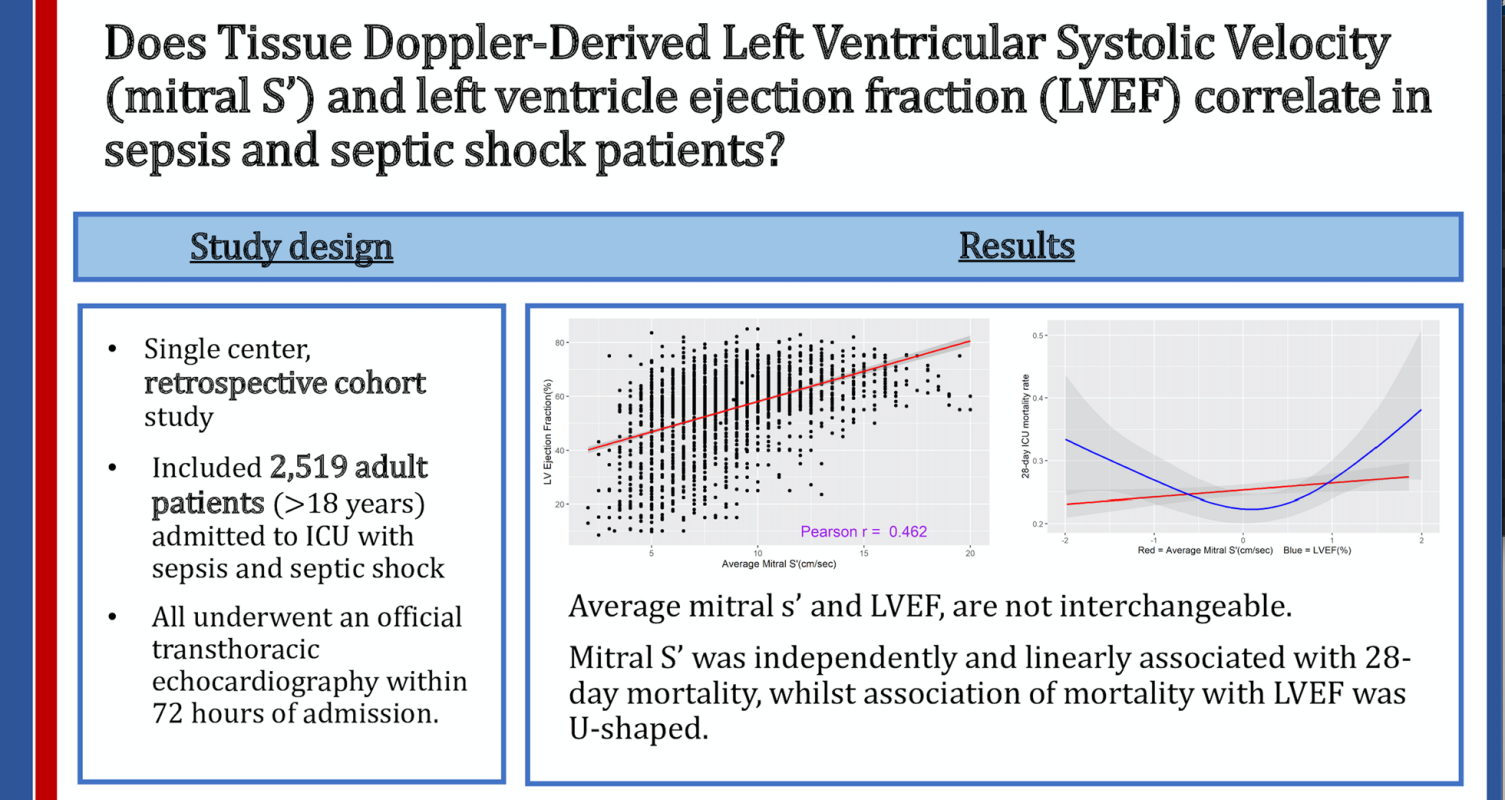

**Supplementary Information:**

The online version contains supplementary material available at 10.1186/s40560-023-00678-z.

## Background

Sepsis remains one of the leading causes of intensive care unit (ICU) mortality in the United States (U.S.) [1]. Cardiac dysfunction or sepsis-induced cardiomyopathy commonly occurs in this patient population, albeit with little consensus on appropriate definition for sepsis cardiomyopathy [2] and can be seen in up to 40–60% and has been shown to be associated with worse outcomes [3, 4].

Echocardiography is the most commonly utilized non-invasive assessment tool to evaluate cardiac function in critically ill patients. In routine clinical practice, global left ventricular (LV) systolic function is predominantly evaluated with the use of ejection fraction using volumetric methods (EF) [5]. However, quantification of LVEF is largely dependent on image quality with clear myocardial blood tissue interface and adequate endocardial definition, which may be limited in critically ill patients, causing high inter- and intra-observer variability [6]. In recent times, the use of Tissue Doppler Imaging (TDI) has emerged as an alternative measurement tool to quantify global systolic and diastolic LV function [7, 8] by evaluating peak annular myocardial velocities [9]. In addition to the ability to quantify LV systolic function in case of limited imaging, TDI-derived LV systolic velocity (mitral S’) measured at the mitral annulus has been shown to be more sensitive in identifying aberrations in LV systolic function when compared to conventional TTE measures in patients with coronary artery disease [10].

Numerous studies have explored the association between mitral S’ and LVEF and found a good-to-excellent correlation these two variables in non-critically ill patients [6, 11–13]. On the other hand, previous studies in critically ill patients have found varied results between these two echocardiographic variables [14, 15]. These studies were limited by their small sample size, ranging between 45 and 50 patients. In addition, prior observational studies examining outcomes among critically ill septic patients have had contradictory results on the impact of mitral S’ on prognosis [16–20].

Considering the challenges of assessing LVEF in critically ill patients, we conducted a retrospective study investigating if TDI obtained mitral S’ can be used interchangeably with LVEF in patients with sepsis and septic shock. Subsequently, this study also assessed the relationship between mitral S’ and clinical outcomes in sepsis.

## Materials and methods

We conducted a retrospective cohort study at the quaternary medical center from January 1, 2011, to December 31, 2020. This study was approved by Cleveland Clinic Institutional Review Board (IRB 15-1233) with a waiver of informed consent. All adult patients (above 18 years of age) admitted to the medical intensive care unit (ICU) with sepsis and septic shock with a TTE performed within 72 h of admission to the ICU were included. The Third International Consensus Definitions for Sepsis and Septic Shock (Sepsis-3) criteria were fitted retrospectively in all patients to define sepsis and septic shock. Septic shock was clinically identified as any patient with sepsis and persistent hypotension requiring initiation of vasopressor medications to maintain a mean arterial pressure (MAP) above 65 mmHg or serum lactate > 2 mmol/L (18 mg/dL) [21]. We excluded any patients (1) less than 18 years of age; (2) had an echocardiogram performed after 72 h after ICU admission; (3) patients without LVEF or mitral S’ (either septal or lateral) measurements; (4) patients with severe mitral and aortic valvular regurgitation; (5) patients with prior prosthetic heart valves or heart transplant; and (6) moderate-to-large pericardial effusion.

The primary outcome was to investigate the relationship between mitral S’ and LVEF. Subgroup analyses of LVEF and mitral S’ correlation were also performed for sex, body mass index (BMI), presence of septic shock, and presence of cardiac dysfunction (defined as LVEF < 45%). Secondary outcomes were the association between mitral S’ and 28-day mortality (counted from ICU admission), hospital-free days, and in-hospital mortality. Hospital-free days were calculated as a composite outcome combining hospital mortality and hospital length of stay, which is calculated as “28—the length of hospital stay” during the first 28 days [22].

All the baseline characteristics and clinical outcome information were obtained retrospectively from patients’ hospital charts utilizing electronic medical records (EMR). The norepinephrine equivalent dose (NEE) was collected using the sum of the norepinephrine equivalent infusion rates of all other inotropic and vasopressor medications administered within 24 h of shock onset. Formula = “norepinephrine + epinephrine + phenylephrine/10 + dopamine/150 + vasopressin × 2.5 (all in µg/kg/min except vasopressin in units/min)” [23, 24].

TTE was performed at the discretion of the primary treatment team. All two-dimensional echocardiograms were performed by experienced sonographers using commercially available ultrasound systems. All echocardiographic measurement was performed offline by experienced professionals utilizing Syngo Dynamics (Siemens Healthcare, MA, USA). To assess LV systolic function, the Simpson method in the apical four- and/or two-chamber views were predominately used to estimate EF in patients as per the American Society of Echocardiography (ASE) recommendations [5]. When endocardial views were not satisfactory or not available, LVEF was visually estimated by qualified cardiologists. TDI was utilized to calculate the peak systolic (s’) wave velocity of the lateral and septal mitral annulus in four-chamber apical views [8]. We measured at least 3 discrete lateral and septal mitral S’ measurements and averaged value was utilized for our analysis, when available. The average mitral S’ was utilized for our final analysis as it was shown in previous studies to better account for differences seen in lateral and septal measurement due to regional wall motion abnormalities [16–18, 20]. As the study has retrospective design we cannot be fully compliant with the items recommended by the PRICES guideline for echocardiography based study in criticall ill [25].

To assess the interrater reliability of the mitral S’ measurements, we randomly selected 20% of the total patient population, and two trained physicians calculated the mitral S’ on two separate occasions under the same basal conditions. Both physicians were blinded to the other’s measurements. We then calculated the intraclass correlation coefficient (ICC) using a two-way random effects model defined by absolute agreement definition [26]. The ICC values were used to evaluate the reliability of the measurements, with values less than 0.5 indicating poor reliability, values between 0.5 and 0.75 suggesting moderate reliability, values between 0.75 and 0.9 pointing towards good reliability, and values greater than 0.90 confirming excellent reliability. In addition, we calculated the Minimal Detectable Change (MDC) to determine the smallest amount of change in the mitral S’ that can be detected with a certain level of confidence [27]. The MDC was calculated using the standard error of measurement (SEM), which represents the average amount of error in the measurements. The SEM was used in conjunction with the critical value of the standard normal distribution at the 95% confidence level to calculate the MDC.

Continuous variables were expressed as a median value with an interquartile range (IQR: 25th and 75th percentiles). Categorical variables were represented as frequencies and percentages. The Pearson correlation test was used to assess the correlation between average mitral S’ to LVEF in all patients. The following *r* values were used to quantify the scale of correlation: *r* < 0.19 = very low, *r* 0.2–0.39 = low, *r* 0.4–0.59 = moderate, *r* 0.6–0.79 = high and r 0.8–1.0 = very high correlation [28]. We a priori decided to perform subgroup analysis on the correlation between LVEF and mitral S’ based on LVEF, BMI, gender, method of LVEF assessment (visual estimate or Simpson calculation) and severity of sepsis to detect any correlation between mitral S’ and LVEF as suggested by prior studies.

Multivariable logistic regression analyses were performed to calculate the odds ratio (OR) of LVEF and mitral S’ for 28-day mortality from ICU admission and overall in-hospital mortality. The logistic regression model was adjusted for a priori determined demographic and clinical relevant variables: age, sex, Acute Physiology And Chronic Health Evaluation (APACHE) III, history of end-stage renal disease on chronic dialysis, cirrhosis, chronic obstructive lung disease, diabetes, immunosuppression, history of malignancy, heart rate at the time of echocardiogram, BMI, non-linear LVEF [29], total intravenous fluid administration on the day of TTE, and total NEE within 24 h of shock onset. Missing values were imputed using the MissForest package, an iterative nonparametric imputation method based on a random forest as we considered missing data to be missing at random. *p* value < 0.05 was considered statistically significant. All statistical analyses were performed using R software version 4.2.3. for Mac (R Development Core Team).

## Results

During the period of study, 3151 patients who were admitted to MICU with sepsis and septic shock underwent TTE within 72 h of admission to the MICU. Of these, 632 (20%) eligible patients were further excluded due to their inability to obtain mitral S’ measurements. Our final study population included 2,519 patients (Additional file 1: Fig. S1). The study population included 1,216 (48.3%) males. The median age of study population was 64 (IQR: 53, 73), and a median APACHE III score of 85 (IQR: 67,108). Further baseline characteristics are detailed in Table 1. The median maximum NEE dose in the first 24 h was 0.33 mcg/kg/min (IQR: 0.15, 0.69). 28-day mortality was 20.7% (522/2519) and in-hospital mortality was 28.3% (712/2519). The median LVEF for the cohort was calculated as 58% (IQR: 50, 65) (Table 1). The median septal, lateral, and average mitral S’ were 8 cm/s (IQR: 6.0, 10.0), 9 cm/s (IQR: 6.0, 10.0), and 8.5 cm/s (IQR: 6.5, 10.5), respectively. The overall fluid balance on the day of TTE was positive of 1171 mL (494–2383).

**Table 1:** Basic demographics, echocardiographic findings and outcomes of all patients

Demographics of the patients (*n* = 2519)		
Age (Years)	Available data (*N*)	64 (53–73)
Male sex		1216 (48.3%)
Body mass index (kg/m^2^)	2314	28.1 (23.5–34.1)
Cirrhosis *n* (%)		340 (13.5%)
COPD *n* (%)		610 (24.2%)
Diabetes Mellitus *n* (%)		800 (31.8%)
Chronic dialysis *n* (%)		348 (13.8%)
Immunosuppression *n* (%)		661 (26.3%)
Malignancy *n* (%)		565 (22.4%)
APACHE III score	2515	85 (67–108)
Type of sepsis		
Sepsis *n* (%)		1486 (59%)
Septic shock *n* (%)		1032 (41%)
Vitals and Labs on ICU admission		
WBC (k/µl)	2384	13.5 [6.5–20.1)
Lactate max in 24 h (mmol/L)	2347	1.90 (0.80–3.80)
Peak troponin (day 7) (ng/dL)		0.01 (0.00–0.12)
Echocardiographic variables		
Systolic blood pressure (mm Hg)	2304	105 (94–1190
Diastolic blood pressure (mm Hg)	2304	56 (51–63)
Heart rate (bpm)	2389	90 977–105)
Fluid balance on the day of echo (ml)	2451	1,171 (494–2,383)
LVEF (%)		58 (50–65)
LVEF assessed by Simpson Method		1785 (70.1%)
LVEDV (ml)	1930	35.93 (25.4–51.8)
LVESV (ml)	1827	95.1 (74.2–120.4)
MV Annulus peak s’ lateral (cm/s)		9.0 (7.0–11.0)
MV Annulus peak s’ septal (cm/s)		8.0 (6.0–10.0)
MV Annulus peak s’ average (cm/s)		8.5 (6.5–10.5)
MV Annulus peak e’ lateral (cm/s)		10.0 (7.0–12.0)
MV Annulus peak e’ septal (cm/s)		7.1 (6.0–9.0)
E/e’ ratio (cm/s)	1878	10.1 (7.7–13.1)
E/A	1790	1.04 (0.78–1.38)
LA volume index (ml/m^2^)	1624	26.2 (18.8–35.7)
TAPSE (cm)	1817	1.90 (1.51–2.30)
RV TDI S’(cm/s)	2314	12.3 (10–15.4)
RVSP (mmHg)	1983	39 (31–48)
LVOT–VTI SV (ml)	2289	65 (44.6–87.2)
LV Cardiac Output (L/min)	2281	5.8 (3.6–9.0)
Time to Echo from ICU admission (Hr)		17 (9–34)
Outcomes		
Max NEE in 24 h (mcg/kg/min)		0.33 (0.15–0.69)
Epinephrine (*n*, %)		221 (8.8%)
Other Inotropes^#^ (*n*, %)		120 (4.7%)
Mechanical ventilation		1228 (48.7%)
28-day mortality from ICU admission		648 (25.7%)
In-hospital mortality		712 (28.3%)
30-day hospital free days		1.97 (0.01–6.59)

Data for the variable available in all patient (*n* = 2519) unless number provided

*COPD* chronic obstructive pulmonary disease, *WBC* white blood cell, *LVEF* left ventricle ejection fraction, *LVEDV* left ventricle end-diastolic volume, *LVESV* left ventricle end-systolic volume, *E/e’* ratio between mitral E wave and tissue Doppler e’ wave, *E/A* ratio between mitral E wave and A wave, *LA* left atrium, *TAPSE* tricuspid annular plane systolic excursion, *RV TDI S’* tricuspid annular peak systolic velocity, *RVSP* right ventricle systolic pressure, *LVOT–VTI* left ventricle outflow tract–velocity time integral, *SV* stroke volume, *LV* left ventricle, *NEE* norepinephrine equivalent dose,

^#^Includes dopamine, dobutamine and milrinone

**Fig. 1:**
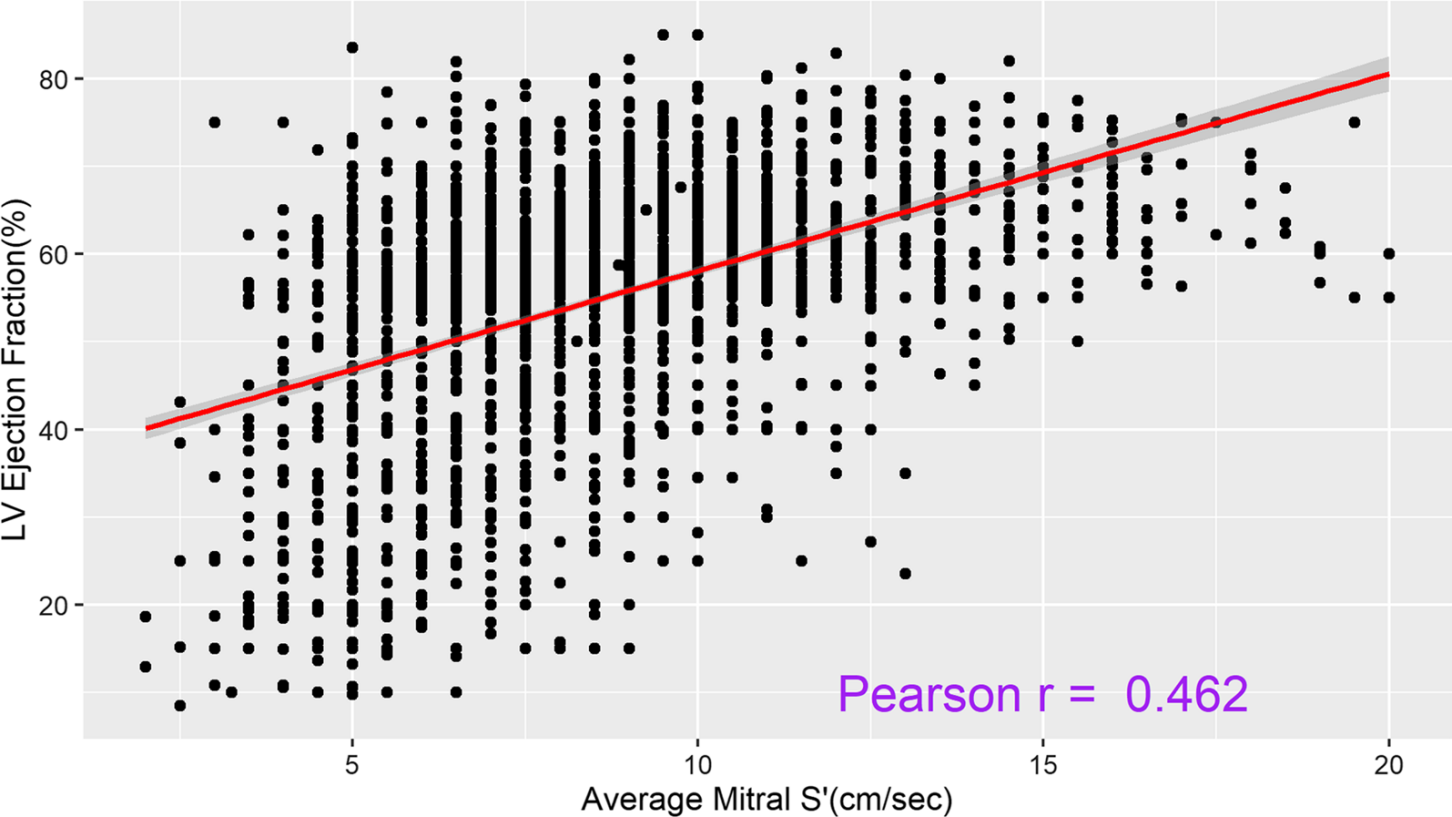
Pearson correlation: left ventricle ejection fraction (LVEF %) and Average Tissue Doppler-Derived Left Ventricular Systolic Velocity (mitral S’) (cm/s)

**Fig. 2:**
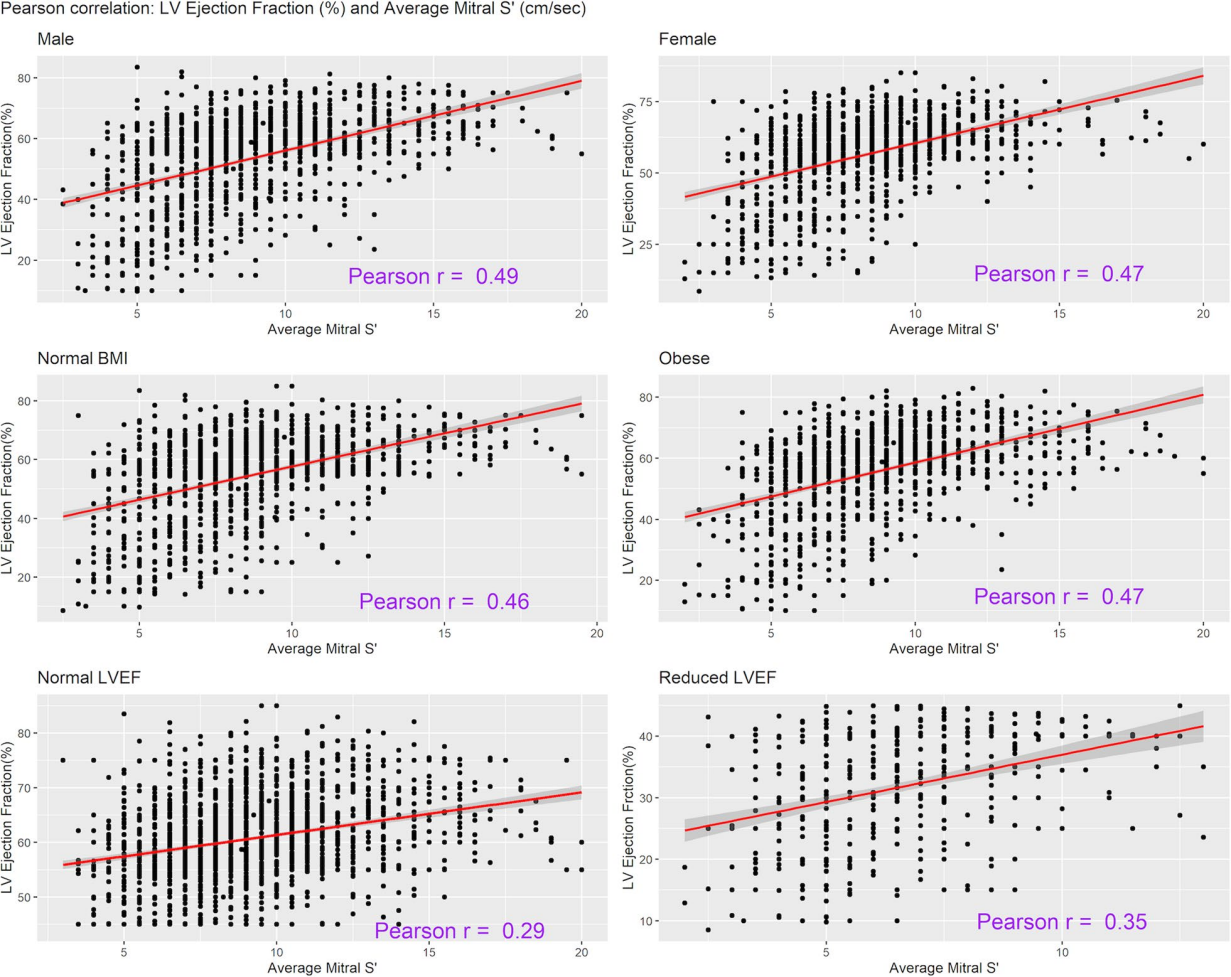
Pearson correlation: left ventricle ejection fraction (LVEF %) and average Tissue Doppler-Derived Left Ventricular Systolic Velocity (mitral S’) (cm/s) in with sub-group analysis based on gender, BMI (BMI < 30 kg/m^2^ or ≥ 30 kg/m^2^), and normal or reduced LVEF (LVEF < 45% or LVEF ≥ 45%)

The Pearson coefficient between LVEF and mitral S’ showed significant correlation but of moderate positive degree only (*r* = 0.46; *p* < 0.001) (Fig. 1). In predefined subgroup analyses, the correlation was 0.35 among patients with reduced LVEF (< 45%) and 0.29 in patients with preserved LVEF (*p* < 0.001) (Fig. 2). In addition, the separation according to gender did not change the strength of the correlation, with correlation coefficient being 0.49 for males and 0.47 for females (*p* < 0.001). The correlation coefficient was 0.47 and 0.46 for patients with normal BMI or obesity, respectively (*p* < 0.001). The correlation coefficient was 0.48 for the LVEF calculated by Simpson method and 0.39 for visually assessed LVEF (*p* < 0.001). Similarly, the correlation coefficient in patients with sepsis, septic shock at low NEE (0–0.5 mcg/kg/min), and septic shock with high NEE (> 0.5 mcg/kg/min) were 0.44, 0.47 and 0.50, respectively (all *p* < 0.001) (Additional file 1: Table S1 and Figure S2). The correlation coefficient between average mitral S’ and average E/e’ (non-invasive filling pressure) and maximum 24 h NEE dose were − 0.41 and − 0.03, respectively (Additional file 1: Figures S3 and S4).

Additional file 1: Table S2 summarizes the interrater reliability of the mitral S’ measurements. The ICC for mitral S’ lateral was 0.625 [95% confidence interval (CI) 0.531–0.701], with an SEM of 0.018 and an MDC at a 95% confidence level of 0.015. For mitral S’ septal, the ICC was 0.936 (95% CI 0.920–0.949), with an SEM of 0.007 and an MDC at a 95% confidence level of 0.02. The ICC for mitral S’ average was 0.808 (95% CI 0.757–0.848), with an SEM of 0.011 and an MDC at a 95% confidence level of 0.033. The *p* values for all ICCs were less than 0.0005, indicating statistical significance. These findings suggest good-to-excellent interrater reliability.

**Fig. 3:**
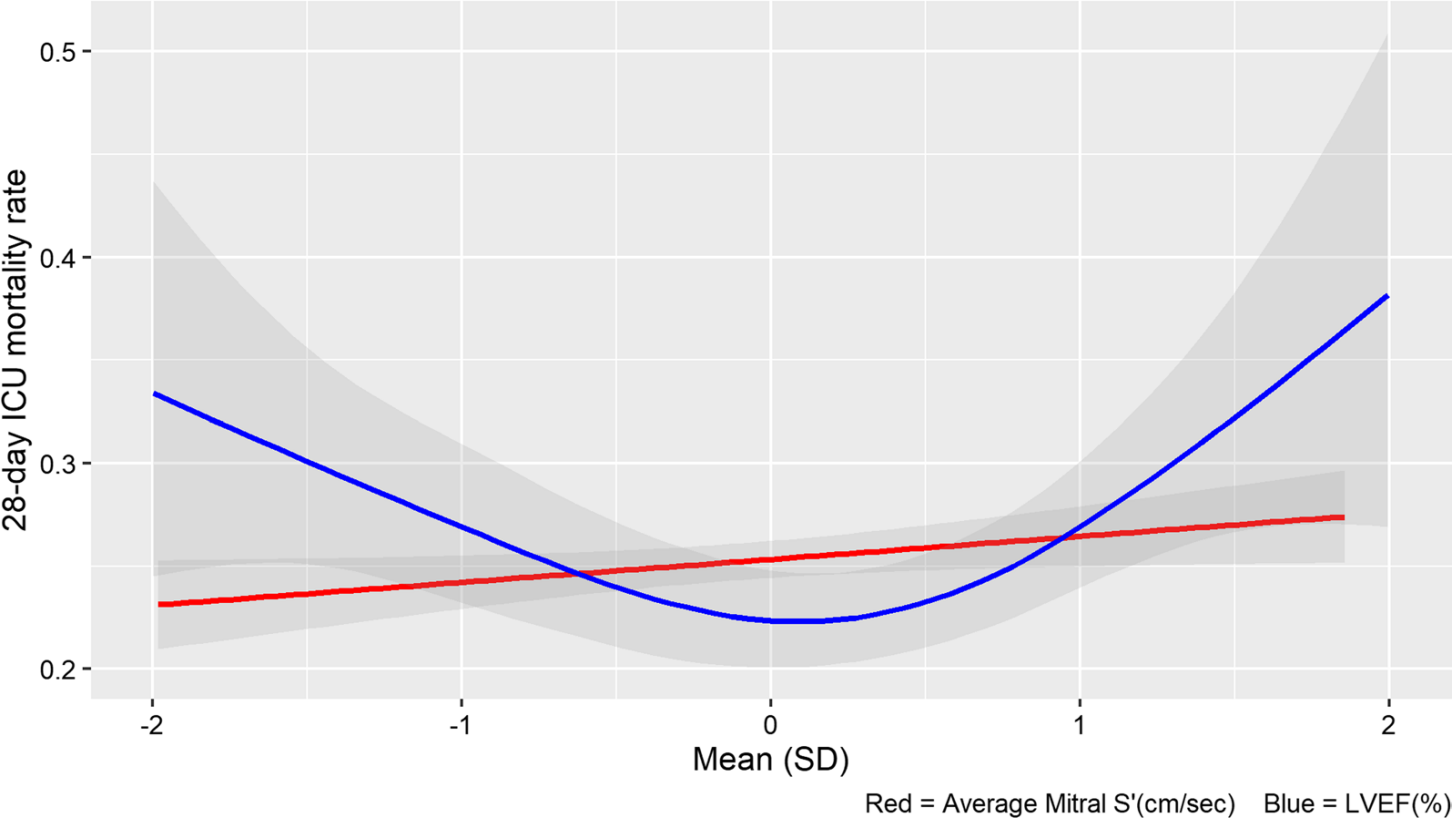
28-day mortality rate and its association with average Tissue Doppler-Derived Left Ventricular Systolic Velocity (mitral S’) and Left ventricle ejection fraction (LVEF %) in patients with sepsis and septic shock

In the multivariable logistic regression analysis conducted after adjusting for LVEF as non-linear variable and for other clinically important variables, we found that higher values of average mitral S’ were associated with an increase of 28-day mortality with OR 1.07 (95% CI 1.02–1.12, *p* = 0.006) as well as with in-hospital mortality with OR 1.07 (95% CI 1.02–1.12, *p* = 0.004) (Table 2 and Additional file 1: Table S3). The relationship between predicted 28-day mortality and average mitral S’ was linear (Fig. 3). In contrast, the relationship between 28-day mortality and LVEF was U-shaped.

**Table 2:** Multivariable logistic regression model for 28-day ICU mortality in patients with sepsis and septic shock

Variables	OR	95% CI	*p* value
Mitral S’ average	1.07	1.02–1.12	0.006
S (LVEF)	–	*–*	< 0.001
Sex (male)	1.00	0.80–1.25	0.987
Age	1.01	1.01–1.02	< 0.001
APACHE III score	1.02	1.01–1.02	< 0.001
BMI	1.01	1.00–1.02	0.130
Cirrhosis	1.61	1.18–2.20	0.002
COPD	1.08	0.84–1.38	0.556
Diabetes mellitus	0.89	0.70–1.13	0.334
Chronic dialysis	1.48	1.08–2.00	0.013
Malignancy	1.67	1.25–2.24	0.001
Immunosuppression	1.07	0.81–1.42	0.616
Heart rate at time of Echo	1.00	0.99–1.00	0.153
Mechanical ventilation	1.80	1.40–2.32	< 0.001
Maximum Lactate in 24 h of shock	1.13	1.09–1.18	< 0.001
Total IV fluid balance on ECHO Day	1.00	1.00–1.00	0.454
Maximum NEE dose in 24 h	2.65	2.05–3.44	< 0.001

*BMI* body mass index, *COPD* chronic obstructive pulmonary disease, *NEE* norepinephrine equivalent dose, *OR* odds ratio, *CI* confidence interval

## Discussion

To the best of our knowledge, we conducted the largest study investigating the correlation between mitral S’ and LVEF in a population of critically ill patients. Our study had two important findings. First, we found only a moderate correlation between mitral S’ and LVEF among patients admitted to the medical ICU with sepsis or septic shock, and this finding was consistent in several prespecified subgroup analyses. Second, mitral S’ was independently associated with a linear increase in 28-day mortality from ICU admission in septic patients, therefore suggesting a statistically significant prognostic value. Conversely, the relationship between LVEF and 28-day mortality from ICU admission was U-shaped.

Cardiac function assessment is integral to the management of sepsis and septic shock. LVEF using the Simpson method is routinely used to assess LV systolic function parameter. However, LVEF measurement often requires an optimal image for measurement, which may be limited in critically ill patients. Mitral annular plane systolic excursion (MAPSE) and TDI-derived LV systolic velocity (mitral S’), both represent regional measurements of LV longitudinal systolic function, have been suggested as good surrogates for the LV systolic function. Both parameters are conceptually simple, do not rely on geometric assumptions, are easy to obtain and highly reproducible even when performed by practitioners with limited experience [30]. Studies have found mitral S’ and MAPSE to have excellent correlation and concordance. Mitral S’ is routinely performed as a part of systolic assessment of the LV, while MAPSE is still not part of comprehensive TTE. The correlation between mitral S’ and LVEF is good-to-excellent among stable cardiac outpatients [6, 11–13, 31]. However, studies in critically ill patients remains limited in sample size, and evidence so far suggests that mitral S’ values are not associated with prognosis in septic patients [14, 15], which was confirmed by a recent meta-analysis [32]. Such meta-analysis included 13 studies and 1200 patient, which is less than half of our sample size. In addition, the sample size varied in the included studies (from 21 to 262). The larger sample size in our study provided more power to detect any relationship between mitral S’ and mortality, and this is a possible explanation for different findings.

Surprisingly, even though mitral S’ has been repeatedly promoted as a good surrogate for LVEF, our study showed these parameters cannot be used interchangeably in critically ill septic patients. We just found a moderate correlation between mitral S’ and LVEF, findings that were consistent across different subgroup analyses with correlation ranging between 0.29 (those with LVEF > 45%) and 0.50 (septic shock with high dose of NEE). The correlation of mitral S’ was low regardless the values of LVEF. The mitral S’ values in reduced LVEF group were lower compared to normal LVEF group, however utility of mitral S’ to diagnose sepsis cardiomyopathy remains limited. These results are comparable to a smaller prospective study by Bergenzaun et al. of 50 patients with septic shock, who underwent TTE every 24 h until 7 days or death with an overall correlation of *r* = 0.473 [14]. Similarly, Furian et al. also demonstrated a moderate correlation (*r* = 0.49; *p* = 0.003) among 45 patients with severe sepsis [15]. Our larger cohort with varied severity and co-morbidities not only validates but conclusively proves that mitral S’ and LVEF are non-interchange entities among critically ill patients.

The moderate correlation between LVEF and mitral S’ in septic patients are likely impacted by several factors. LVEF assessment includes both the radial and longitudinal components of LV systolic contraction. Normally, longitudinal shortening contributes approximately 75% to cardiac contractility and overall stroke volume [33]. As short axis shortening (radial function) gets impaired with various disease states, the heart compensates by increasing contribution from the longitudinal component to maintain cardiac function. This adaptation may partially explain why our study and previous evidence demonstrated only a moderate correlation observed between mitral S’ and LVEF. Another explanation is the impact of loading conditions on LVEF and mitral S’. LVEF is often reflective of the coupling between LV contractility and its afterload [34, 35]. Therefore, it is affected by both preload and afterload changes, the latter being particularly reduced in patients with septic shock. Thus, septic patients with reduced intrinsic LV contractility may show a preserved LVEF in the setting of severely reduced afterload [30]. Conversely, mitral S’ seems influenced by afterload to a lesser extent and to depend mostly on changes in preload [36–39]. We observed the LVEF to sepsis mortality curve to be U-shaped, while it increases linearly with mitral S’. We hypothesize the different shape of the curve stems from difference in parameters, which are assessed and we believe these may be mainly due to influence of loading conditions on each of these parameters, especially the afterload that highly affects LVEF and possibly to a lesser extent for s’. For instance, the higher mortality of hyperdynamic LVEF can be attributed to under-resuscitation, severe vasoplegia, and/or sympathetic overstimulation, whilst values of s’ can greatly vary. However, despite this possible physiological interpretation, such hypothesis is not supported by the subgroup analyses where we assessed correlation according to the LVEF and to vasopressor dosages”. Notably, the same moderate correlation was reported by two smaller studies that we discussed. Hence, our findings are in the same direction. The reasons why correlation is lower in critically ill patients as compared to other groups of stable patients deserves prospective evaluation.

We acknowledge that recent data has shown that global longitudinal strain (GLS) can potentially identify early myocardial dysfunction, often missed by the conventional indexes of systolic function [40] (as LVEF). GLS is dependent on LV loading conditions, but the largest influence on this parameter seems due to afterload changes [37, 38, 41–43]. The possibly lower degree of dependence of GLS on preload as compared to LVEF and TDI variables makes GLS an exciting prognostic variable for critically ill patients. However, strain echocardiography is not widely available for clinical use [44]. Mitral S’ has been shown to detect myocardial contraction impairment before clinical deterioration. This would potentially make mitral S’ an attractive alternative to GLS to effectively study the full spectrum of LV systolic dysfunction.

The biggest strength of our study is the large number of patients included in the final analysis and the use of homogeneous criteria for the diagnosis of sepsis and septic shock. We also assessed for intra-operator reliability of mitral S’ measurement to reduce measurement errors influencing our results. However, our study remains a single-center retrospective cohort study, and as a result, we cannot eliminate selection bias completely. Including all consecutive patients who met the study criteria has mitigated some risks of selection bias. Second, the time window for echocardiograms in this study was three days after medical ICU admission. The loading conditions and vasopressor dosage can change significantly during the first three days after ICU admission. Most patients (77.3%) underwent an echocardiogram within 24 h of admission to ICU, coinciding with the onset of sepsis and septic shock. Pulmonary artery catheter, central venous pressure, and central venous oxygen saturation are no longer frequently assessed in routine critical care. The unavailability of more precise loading parameters and markers of tissue perfusion other than MAP, fluid balance, and serum lactate in our sample limits our ability to understand the impact of loading conditions and tissue perfusion on mitral S’ and mortality. The retrospective nature of the study limits also our ability to completely abide by requirements of PRICES guidelines. Another limitation, is the exclusion of patients where peak annular velocity was not available, particularly from the lateral mitral annulus. However, in all patients and subgroup analyses, average mitral S’ had similar or better correlation compared to either lateral or septal velocities (Additional file 1: Table S1).

## Conclusion

In a large study of patients with sepsis and septic shock, we found a moderate correlation between average mitral S’ and LVEF, suggesting these variables are not interchangeable. Mitral S’ was independently and linearly associated with 28-day mortality, whilst association of mortality with LVEF was U-shaped.

## Supplementary Information

**Additional file 1: Table S1. **Pearson correlation between LVEF % and Mitral S’with sub group analyis based on gender, BMI, heart failure and various severity of sepsis. **Table S2.** Assessing interrater reliability for Mitral S’ measurements. **Table S3.** Multivariable logistic regression model for in-hospital mortality in patients with sepsis and septic shock. **Figure S1.** PRISMA flow diagram representing the final study population. **Figure S2.** Pearson Correlation between Average Mitral S’ and LVEF among subgroup analysis. **Figure S3.** Pearson correlation between average Mitral S’ and average E/e’. **Figure S4.** Pearson Correlation between Average Mitral S’ and 24-h Max Norepinephrine Equivalent dose

## Data Availability

The datasets generated and/or analyzed during the current study are not publicly available due to HIPPA violation but are available from the corresponding author on reasonable request.

## Abbreviations

APS: Acute Physiology Score
APACHE III: Acute physiology, age, chronic health evaluation
COPD: Chronic obstructive lung disease
CI: Confidence interval
ICU: Intensive care unit
IQR: Interquartile range
OR: Odds ratio
LVEF: Left ventricular ejection fraction
LVEDD: Left ventricle end-systolic diameter
LVESD: Left ventricle end-diastolic diameter
LVEDV: Left ventricle end-systolic volume
LVESV: Left ventricle end-diastolic volume
Mitral S’: TDI derived peak mitral annular systolic velocity
MICU: Medical intensive care unit
NEE: Norepinephrine equivalent dose
TAPSE: Tricuspid annular plane systolic excursion
TDI: Tissue Doppler imaging
TTE: Transthoracic echocardiogram
RV S’: Tricuspid annulus peak systolic velocity
