# Supplementary material for: Correlation between tissue Doppler-derived left ventricular systolic velocity (S’) and left ventricle ejection fraction in sepsis and septic shock: a retrospective cohort study
Source: J Intensive Care. 2023 Jul 3;11:28. doi: 10.1186/s40560-023-00678-z (PMC10316553; doi:10.1186/s40560-023-00678-z)

**Additional material**

**Additional Table 1: Pearson correlation between LVEF % and Mitral S’ (cm/sec) with sub group analyis based on gender, BMI, heart failure and various severity of sepsis**

| Correlation | Average Mitral S’ | Lateral | Septal |
| --- | --- | --- | --- |
| Male | 0.49 | 0.43 | 0.48 |
| Female | 0.47 | 0.41 | 0.45 |
| Normal (BMI <30) | 0.46 | 0.4 | 0.46 |
| Obese (BMI ≥ 30) | 0.47 | 0.43 | 0.44 |
| Reduced LVEF (LVEF < 45%) | 0.35 | 0.3 | 0.32 |
| Normal LVEF (LVEF ≥ 45%) | 0.29 | 0.26 | 0.27 |
| LVEF assessed by Simpson Method | 0.48 | 0.44 | 0.47 |
| LVEF assessed by Visual Method | 0.39 | 0.35 | 0.4 |
| Sepsis | 0.44 | 0.4 | 0.49 |
| Septic shock (0 – 0.5 mcg/kg/min) | 0.47 | 0.42 | 0.44 |
| Septic shock (> 0.5 mcg/kg/min) | 0.5 | 0.46 | 0.46 |
| All | 0.46 | 0.45 | 0.41 |

**Additional Table 2: Assessing interrater reliability for Mitral S’ measurements:**

|  | **ICC*** | **95% confidence interval** | | **SEM** | **MDC 95** | P value |
| --- | --- | --- | --- | --- | --- | --- |
|  |  | **Lower bound (m/s)** | **Upper bound (m/s)** |  |  |  |
| **Mitral annular S’ Lateral** | 0.625 | 5.31 | 7.01 | 0.018 | 0.051 | <0.001 |
| **Mitral annular S’ Septal** | 0.936 | 9.20 | 9.49 | 0.007 | 0.02 | <0.001 |
| **Mitral annular S’ Average** | 0.808 | 7.57 | 8.48 | 0.011 | 0.033 | <0.001 |

**ICC;** Intraclass correlation coefficients using an absolute agreement definition**, SEM;** Standard Error of Measurement**, MDC 95;** Minimal Detectable Change based on a 95% confidence interval

*Values less than 0.5 are indicative of poor reliability, values between 0.5 and 0.75 indicate moderate reliability, values between 0.75 and 0.9 indicate good reliability, and values greater than 0.90 indicate excellent reliability

**Additional Table 3: Multivariable logistic regression model for in-hospital mortality in patients with sepsis and septic shock**

| **Variables** | **OR** | **95% Confidence interval** | ***p*-value** |
| --- | --- | --- | --- |
| Mitral S’ average | 1.07 | 1.02 – 1.12 | 0.004 |
| S (LVEF) | – | *–* | <0.001 |
| Sex (male) | 1.07 | 0.86 – 1.32 | 0.564 |
| Age | 1.01 | 1.00 – 1.02 | 0.002 |
| APACHE III score | 1.02 | 1.01 – 1.02 | <0.001 |
| BMI | 1.01 | 0.99 – 1.02 | 0.115 |
| Cirrhosis | 1.87 | 1.38 – 2.53 | <0.001 |
| COPD | 1.10 | 0.86 – 1.39 | 0.450 |
| Diabetes Mellitus | 0.93 | 0.73 – 1.17 | 0.522 |
| Chronic dialysis | 1.52 | 1.12 – 2.04 | 0.006 |
| Malignancy | 1.62 | 1.21 – 2.15 | 0.001 |
| Immunosuppression | 1.16 | 0.88 – 1.51 | 0.295 |
| Heart rate at time of Echo | 1.00 | 0.99 – 1.00 | 0.139 |
| Mechanical ventilation | 2.01 | 1.58 – 2.56 | <0.001 |
| Maximum Lactate in 24 hours of shock | 1.12 | 1.09 – 1.16 | <0.001 |
| Total IV fluid balance on ECHO Day | 1.00 | 1.00 – 1.00 | 0.466 |
| Maximum NEE dose in 24 hours | 2.68 | 2.07– 3.50 | <0.001 |

**Additional figure 1: PRISMA flow diagram representing the final study population**


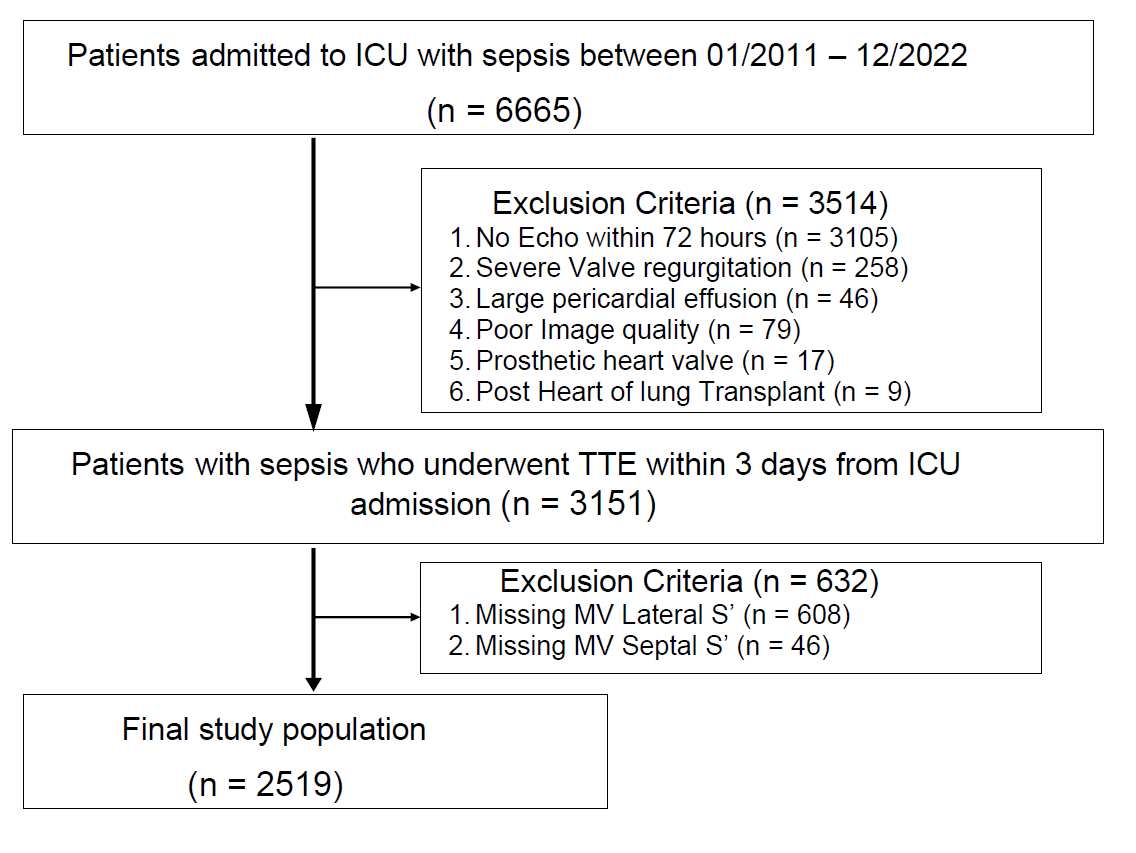


**Additional figure 2: Pearson Correlation between Average Mitral S’ and LVEF among subgroup analysis.**


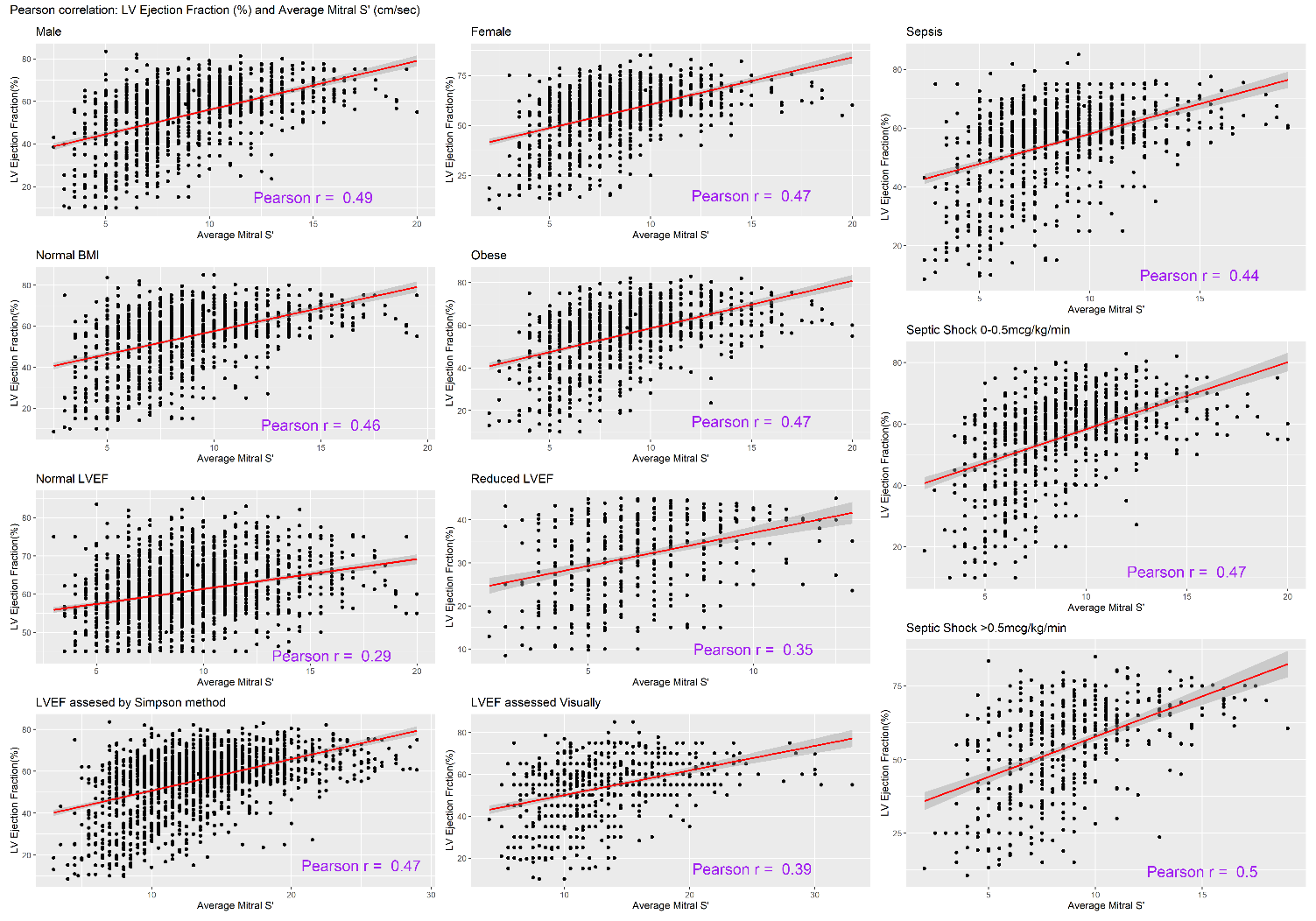


**Additional figure 3: Pearson correlation between average Mitral S’ and average E/e’**


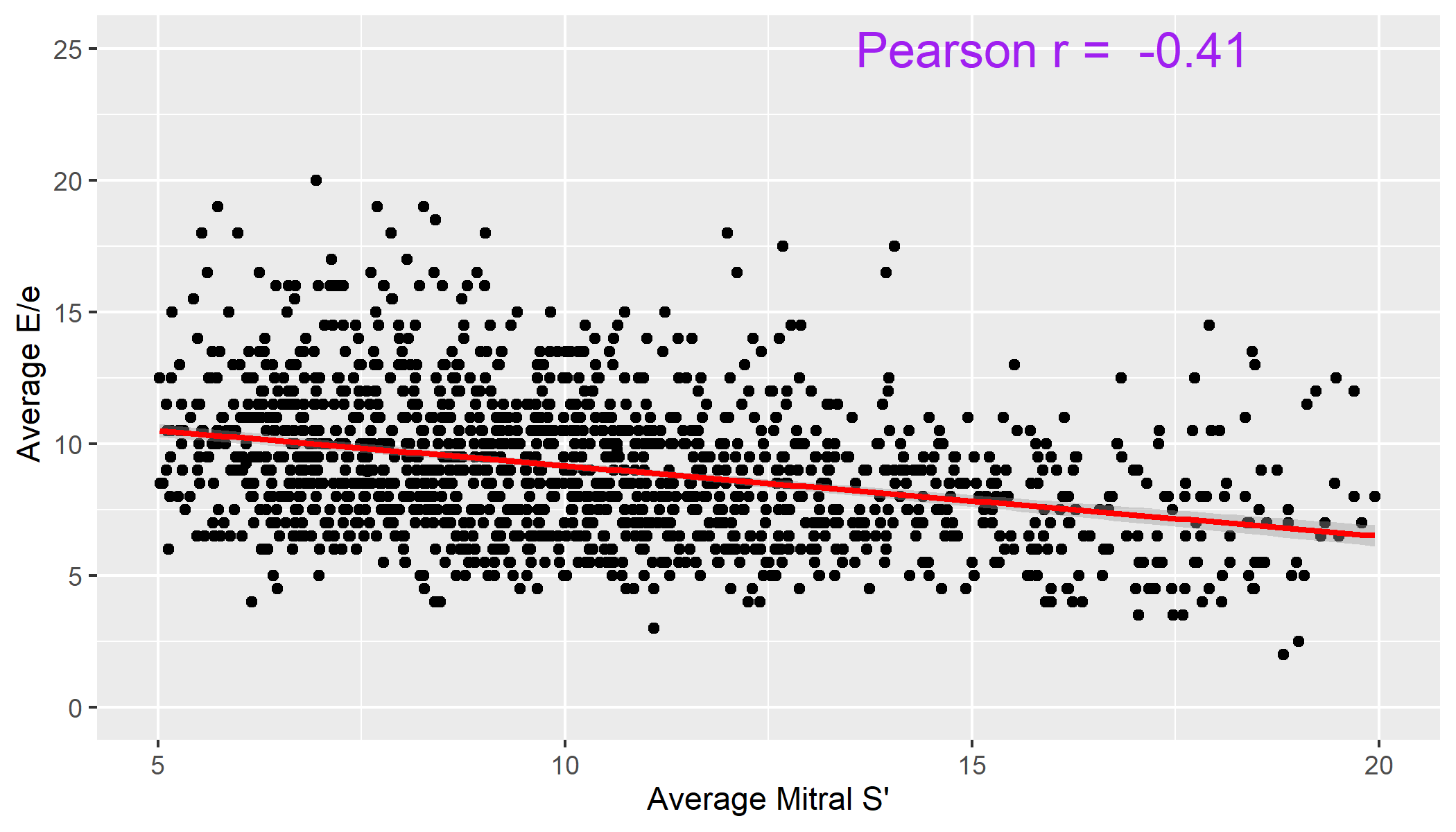


**Additional figure 4: Pearson Correlation between Average Mitral S’ and 24-hour Max Norepinephrine Equivalent dose**


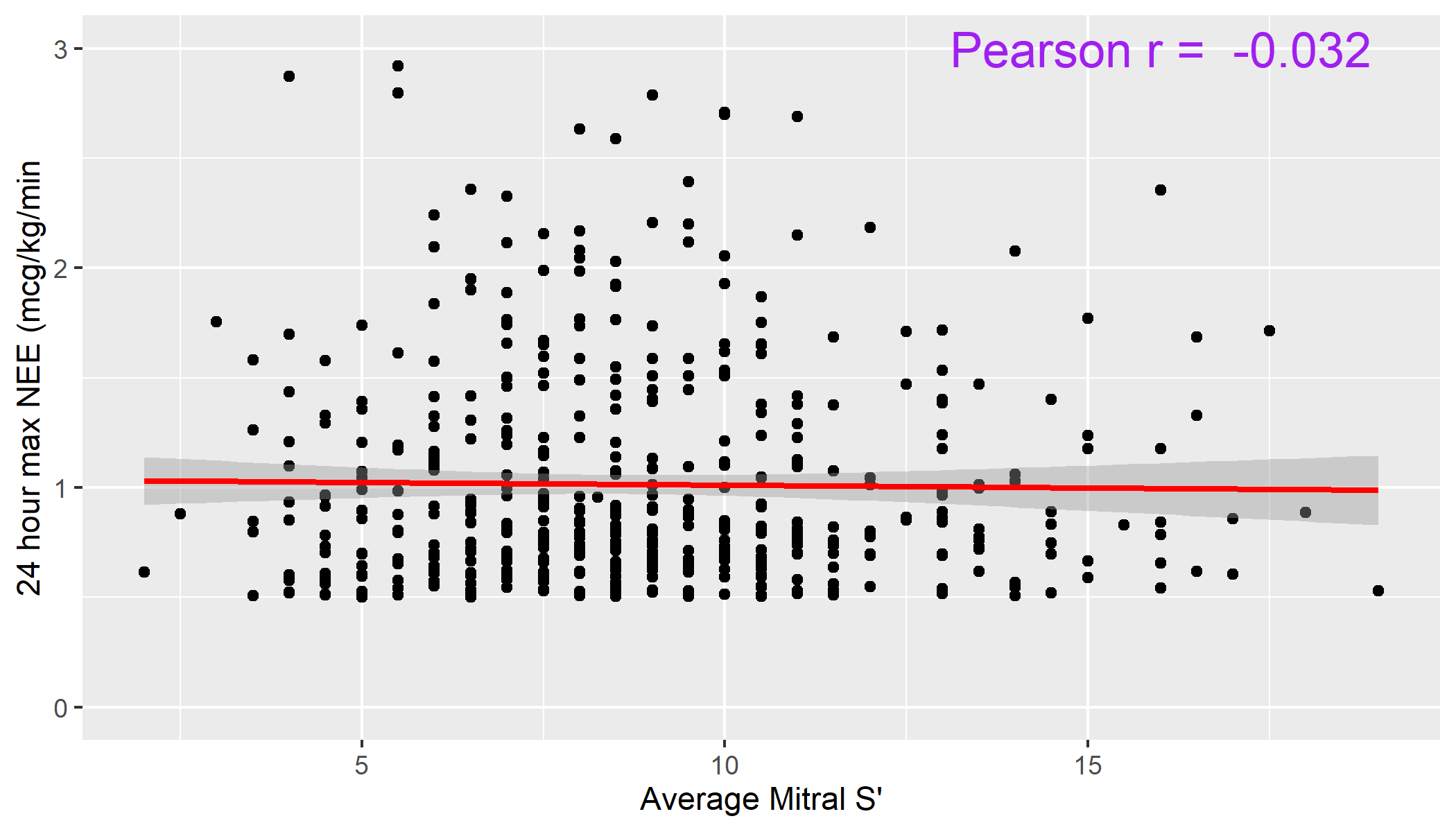

Supplement: Supplementary file 1 — Additional file 1: Table S1. Pearson correlation between LVEF % and Mitral S’with sub group analyis based on gender, BMI, heart failure and various severity of sepsis. Table S2. Assessing interrater reliability for Mitral S’ measurements. Table S3. Multivariable logistic regression model for in-hospital mortality in patients with sepsis and septic shock. Figure S1. PRISMA flow diagram representing the final study population. Figure S2. Pearson Correlation between Average Mitral S’ and LVEF among subgroup analysis. Figure S3. Pearson correlation between average Mitral S’ and average E/e’. Figure S4. Pearson Correlation between Average Mitral S’ and 24-h Max Norepinephrine Equivalent dose [file 40560_2023_678_MOESM1_ESM.docx]
